# Co-Infection with *Mycobacterium tuberculosis* Impairs HIV-Specific CD8+ and CD4+ T Cell Functionality

**DOI:** 10.1371/journal.pone.0118654

**Published:** 2015-03-17

**Authors:** Shivan Chetty, Pamla Govender, Jennifer Zupkosky, Mona Pillay, Musie Ghebremichael, Mahomed-Yunus S. Moosa, Thumbi Ndung’u, Filippos Porichis, Victoria O. Kasprowicz

**Affiliations:** 1 HIV Pathogenesis Programme, Doris Duke Medical Research Institute, University of KwaZulu-Natal, Durban, South Africa; 2 KwaZulu-Natal Research Institute for Tuberculosis and HIV (K-RITH), University of KwaZulu-Natal, Durban, South Africa; 3 The Ragon Institute of MGH, MIT and Harvard, Cambridge, Massachusetts, United States of America; 4 Department of Infectious Disease, Division of Internal Medicine, Nelson R. Mandela School of Medicine, University of KwaZulu-Natal, Durban, South Africa; 5 Max Planck Institute for Infection Biology, Berlin, Germany; Institute of Infection and Global Health, UNITED KINGDOM

## Abstract

The ability of antigen-specific T cells to simultaneously produce multiple cytokines is thought to correlate with the functional capacity and efficacy of T cells. These ‘polyfunctional’ T cells have been associated with control of HIV. We aimed to assess the impact of co-infection with *Mycobacterium tuberculosis* (MTB) on HIV-specific CD8+ and CD4+ T cell function. We assessed T cell functionality in 34 South African adults by investigating the IFN-y, IL-2, TNF-α, IL-21 and IL-17 cytokine secretion capacity, using polychromatic flow cytometry, following HIV Gag-specific stimulation of peripheral blood mononuclear cells. We show that MTB is associated with lower HIV-specific T cell function in co-infected as compared to HIV mono-infected individuals. This decline in function was greatest in co-infection with active Tuberculosis (TB) compared to co-infection with latent MTB (LTBI), suggesting that mycobacterial load may contribute to this loss of function. The described impact of MTB on HIV-specific T cell function may be a mechanism for increased HIV disease progression in co-infected subjects as functionally impaired T cells may be less able to control HIV.

## Introduction

HIV and Tuberculosis (TB) are severe global dual-epidemics. Data suggest that co-infection with HIV and *Mycobacterium tuberculosis* (MTB) increases disease progression of both diseases[1]. For example, higher HIV viral loads are observed in MTB co-infection and increased HIV replication occurs in MTB infected macrophages [2, 3]. The high levels of inflammation and immune activation, as present in TB, may create an optimal cytokine milieu for HIV replication[4]. Whilst immunological impairment is likely to contribute to the increased morbidity and mortality associated with co-infection, the specific mechanisms remain largely unknown. Several studies have reported an impact of HIV on MTB-specific T cell immunity [5,6, 7]. For example, increased infection and lysis of MTB-specific T cells has been accredited to HIV infection [5, 6]. Day *et al* showed that HIV infection impairs MTB-specific responses in HIV co-infection with LTBI, demonstrating that the proportion of IL-2 secreting MTB-specific CD4+ T cells inversely correlated with HIV viral load [7].

The ability of antigen-specific T cells to simultaneously produce multiple cytokines is believed to correlate with the functional capacity and efficacy of T cells. Frequency of these ‘polyfunctional’ T cells in blood samples from infected subjects has been associated with clinical control of HIV and TB [8, 9]. For example, higher bacterial load has been shown to decrease MTB-specific T cell functionality and mono-functional T cells have been shown to dominate functionality profiles in TB as compared to LTBI [10]. Harari *et al* have reported that greater proportions of TNF-α single-positive CD4 T cells are present in individuals with active TB as compared with LTBI [9]. If and how MTB co-infection affects HIV-specific T cell function and polyfunctionality is unknown.

## Methods

### Participants and Study Samples

We enrolled 13 HIV positive individuals with active TB, 9 HIV positive individuals with latent MTB (LTBI), and 11 HIV positive individuals without evidence of LTBI or active TB (Table 1). All were chronically infected HIV positive South-African adults and were CD4 T cell count matched. Viral loads did not significantly differ between patient groups (p = 0.978). TB was identified by a positive sputum acid-fast bacillus smear or sputum culture. LTBI was defined as a positive ESAT-6/CFP-10 IFN-gamma ELISPOT, in the absence of signs and symptoms of TB [11]. Ethical approval and written informed consent from participants was obtained (University of KwaZulu-Natal Biomedical Research Ethics Committee: E028/99 and H020/06). Patients were anti-retroviral treatment naive and not receiving anti-TB treatment.

**Table 1:** Viral load and CD4 count information for study participants.

HIV+				HIV+/LTBI				HIV+/TB			
Patient ID.	CD4 Count (cells/ul)	Viral Load	Sex	Patient ID.	CD4 Count (cells/ul)	Viral Load	Sex	Patient ID.	CD4+ count (cells/ul)	Viral Load	Sex
SK 010 B21	218	5822	F	SK 141 B20	278	2676	F	PID 1079	209	2900	M
SK 114 B14	274	29100	F	SK 195 B20	207	10077	M	PID 1120	460	3700	M
SK 139 B20	184	47547	F	SK 236 B9	184	272227	F	PID 1135	253	470000	M
SK 142 B12	275	186858	F	SK 351 B15	350	285891	F	PID 1224	345	3850	M
SK 208 B11	126	25523	F	SK 359 B15	466	9449	F	PID 1225	366	224137	M
SK 212 B22	233	19670	F	SK 364 B15	285	3140	M	PID 821	110	20000	F
SK 278 B17	443	26036	F	SK 391 B13	247	1936	F	PID 683	195	430000	F
SK 324 B14	353	144252	F	SK 397 B14	332	198266	M	PID 863	204	49000	M
SK 373 B15	184	58237	F	SK 425 B13	192	101600	F	PID 917	239	66000	F
SK 410 B11	495	2570	F					PID 929	270	540000	M
SK 444 B13	331	21447	F					PID1024	239	88000	M
								PID1046	267	17000	M

### Flow cytometry

We assessed T cell functionality using a multi-parameter flow cytometry panel: Viability marker, CD3, CD4, CD8, IFNγ, IL-2, TNF-α, IL-21 and IL-17. Intracellular cytokine staining (ICS) of peripheral blood mononuclear cells (PBMC) was performed following a 6 hour stimulation with either Staphylococcal enterotoxin B (SEB), an HIV Gag peptide pool, or an MTB-specific ESAT-6/CFP-10 peptide pool. FlowJo (version 8.3.3; Treestar) and GraphPad Prism (V.5.5) software were used to analyze the data. A positive antigen-specific response was defined as greater than or equal to 0.05% of the T cell subset analyzed, and 3 times above background.

### Statistical analysis

GraphPad Prism (V.5.5) was used to perform all statistical analysis. Mann-Whitney test was used to compare continuous outcomes between two groups. For more than two groups comparison, Kruskall-Wallis test with Dunn’s post hoc analyses was used. F Fisher’s exact test was used to compare categorical outcomes (i.e., pie charts). All *p* values are two sided and a p-value<0.05 was considered significant.

## Results

**Fig 1:**
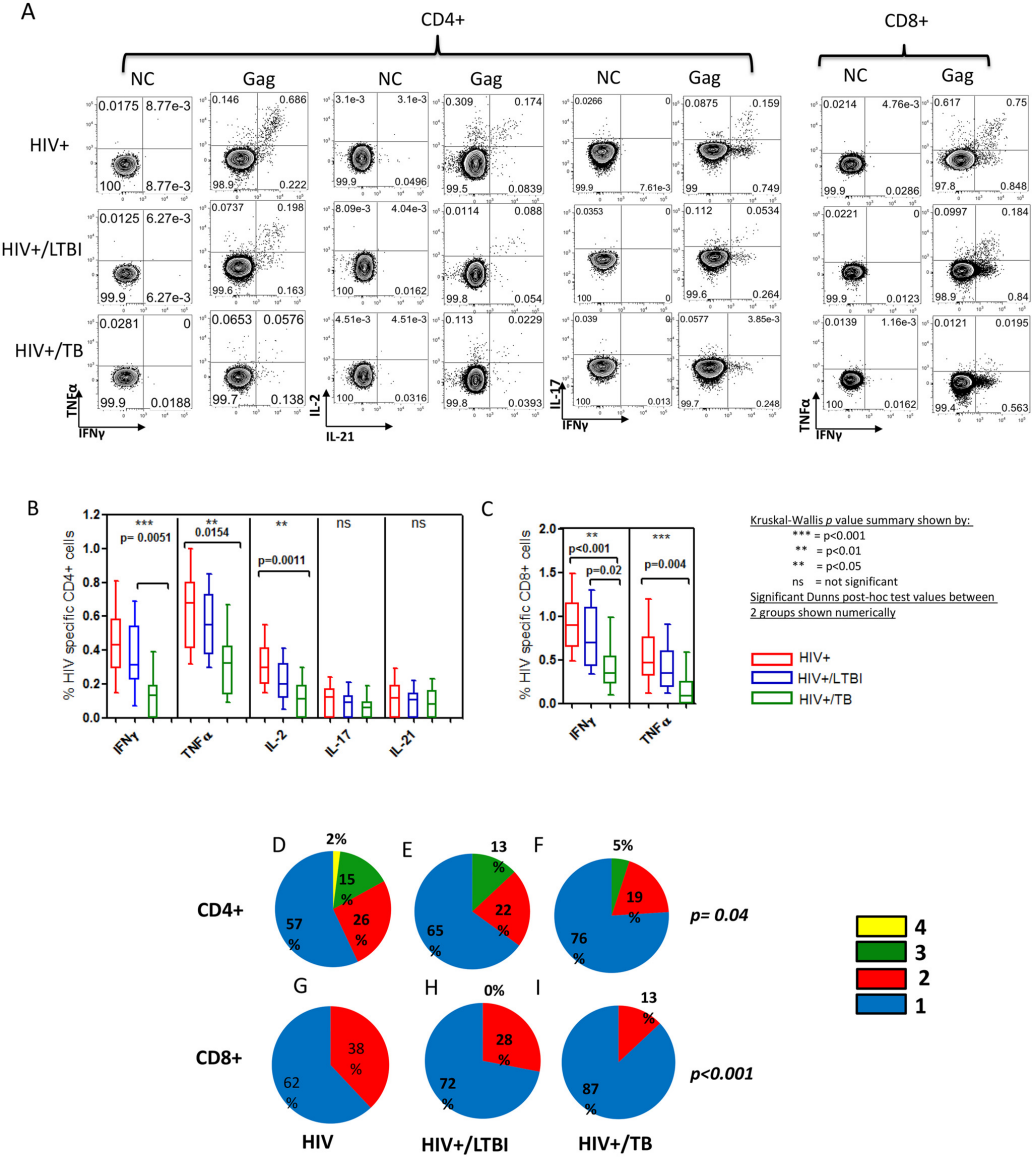
HIV specific CD4+ and CD8+ T cell functionality is reduced in HIV infected individuals co-infected with latent Mycobacterium tuberculosis infection (LTBI) and active Tuberculosis (TB) disease. **(A)** Representative flow cytometry plots showing cytokine responses for HIV (gag), control (no antigen) for HIV mono-infected subjects, and subjects co-infected with latent MTB infection (LTBI) and active tuberculosis (TB) **(B)** HIV-specific CD4+ release of IFNγ (p<0.001), TNFα (p<0.01) and IL-2 (p<0.01) were significantly different between all groups (Kruskal-Wallis). **(C)** HIV-specific CD8+ release of IFNγ (p<0.01) and TNFα (p<0.001) was significantly lower from HIV mono-infected subjects, to those co-infected with active TB. We additionally assessed the polyfunctionality profile of HIV specific CD4+ and CD8+ T cells in all patient groups. The polyfunctionality profiles between groups differed significantly for both CD4+ (p = 0.04) and CD8+ (p<0.001).**(D)** HIV-specific CD4+ T cells from HIV mono-infected subjects displayed a polyfunctional CD4+ T cell profile with a maximum of four functions being present (IFN^+^IL-2^+^IL-17^+^TNFα (2%)).**(E)** HIV-specific four function CD4+ T cells were not present in subjects co-infected with LTBI **(F)** Further decreases in HIV-specific CD4+ T cell polyfunctionality were observed in HIV positive subjects co-infected with TB, being replaced by a largely mono-functional profile with a decreased amount of triple cytokine cells (5% as compared to 13% in HIV/LTBI and 15% in HIV mono-infection). Additionally, single positive TNF-α cells dominated the profile (48%). **(G)** HIV-specific CD8+ T cells in HIV mono-infected subjects displayed a polyfunctional profile with a maximum of 2 functions being present (IFN^+^ TNFα (2%). **(H)** A maximum of 2 functions (IFN^+^TNFα^+^ (28%)) were present in HIV-specific CD8+ cells from subjects co-infected with LTBI. **(I)** 87% mono-functional cells were present in the HIV-specific CD8+T cell profile from subjects co-infected with TB, suggesting a complete loss of polyfunctionality.

HIV-specific CD4 T cells were readily detectable in mono-infected individuals (Fig. 1A and 1B). HIV-specific CD4+ cell release of IFN-γ was significantly lower in HIV+/TB as compared to HIV+/LTBI (p = 0.005)(Fig. 1B). HIV-specific CD4+ cell release of TNF-α (p = 0.01) and IL-2 (p<0.001) were significantly lower in HIV+/TB as compared to HIV mono-infected individuals. In summary, HIV co-infection with MTB was associated with a decrease in the amount of cytokine (IFN-γ, TNF-α and IL-2) secreted for HIV-specific CD4+ T cells.

HIV-specific CD8+ release of IFN-γ (p<0.001) and TNFα (p = 0.004) were significantly lower in HIV/TB co-infected subjects as compared to HIV mono-infected subjects (Fig. 1C). Additionally, IFN-γ secretion was found to be significantly lower in the HIV+/TB group as compared to the HIV+/LTBI group (p = 0.02). No significant antigen-specific production of IL-2, IL-17 or IL-21 was observed for CD8+ T cell responses. In summary, HIV co-infection with MTB was associated with a decrease in the amount of cytokine (TNF-α and IFN-γ) secreted for HIV-specific CD8+ T cells.

Significant differences were also observed between the polyfunctionality profiles for CD4+ (p = 0.04) and CD8+ (p<0.001) T cells (Fig. 1 D-I). HIV mono-infected subjects displayed a polyfunctional HIV-specific CD4+ T cell profile with a maximum of four functions (IFN-γ^+^IL-2^+^IL-17^+^TNF-α^+^ (2% of HIV-specific CD4+ T cells)(Fig. 1d). 15% of HIV-specific CD4 T cells were able to secrete 3 cytokines simultaneously (IFN-γ^+^IL-2^+^TNF-α^+^), 26% secreted 2 cytokines (IFN-γ^+^TNF-α^+^(15%),IFN-γ^+^IL-2^+^(6%), IL-2^+^IL-21^+^(5%))while 57% were mono-functional (IFN-γ^+^(31%), TNF-α^+^(26%)). HIV-specific four-function CD4+ T cells were not present in those co-infected with LTBI and this was accompanied by a higher proportion of mono-functional T cells: 65%(IFN-γ^+^(29%),TNF-α^+^(36%))(Fig. 1E).Further changes in HIV-specific CD4+ T cell polyfunctionality were observed in HIV positive subjects co-infected with TB, with only 5% being able to co-secrete 3 cytokines (IFN-γ^+^IL-2^+^TNF-α^+^) and 76% with mono-functional secretion capacity (TNF-α only (47%), IL-2 only (2%), or IFN-γ only (26%))(Fig. 1G). In summary, HIV co-infection with MTB was associated with a decrease in the number of cytokines secreted from HIV-specific CD4+ T cells.

In HIV mono-infected individuals the HIV-specific CD8+ T cell profile consisted of dual cytokine secreting cells IFN-γ^+^TNF-α^+^ (38% of total HIV-specific CD8+ T cells) and mono-functional cells (62% of total HIV-specific CD8+ T cells)(Fig. 1G).Bi-functional T cells were found to be present at progressively lower frequencies from HIV mono-infection, to co-infection with LTBI, to co-infection with TB (Fig. 1 G-1). 87% mono-functional HIV-specific CD8+ T cells were present in subjects co-infected with TB (Fig. 1I). In summary, HIV co-infection with MTB was associated with a decrease in the number of cytokines secreted from HIV-specific CD8+ T cells.

**Fig 2:**
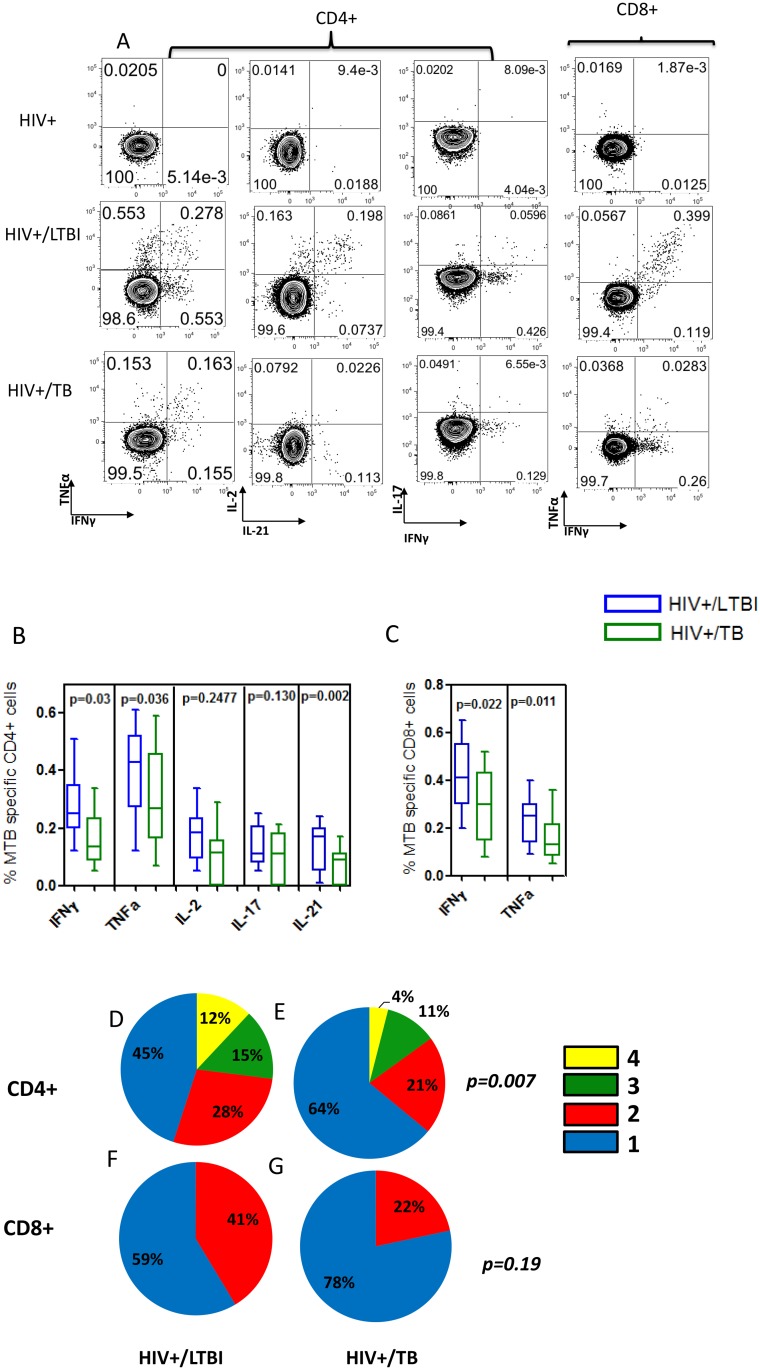
MTB-specific CD4+ and CD8+ T cell functionality is reduced in HIV infected individuals co-infected with latent Mycobacterium tuberculosis infection (LTBI) and active tuberculosis (TB) disease. **(A)** Representative flow cytometry plots showing cytokine IFN-gamma and TNF-α response for Mycobacterium tuberculosis (MTB) (CFP-10 and ESAT-6). Corresponding negative controls (no antigen) are the same as shown in Fig. 1B. **(B)** MTB-specific CD4+ release of IFNγ (p = 0.03), TNFα (p<0.03) and IL-21(p<0.002) were observed to be significantly lower from subjects co-infected with LTBI to those co-infected with TB. **(C)** MTB-specific CD8+ release of IFNγ (p = 0.022) and TNFα (p<0.011), were lower subjects co-infected with TB as compared co-infected with LTBI. We next assessed the polyfunctionality profile of Mycobacterium tuberculosis (MTB)—specific CD4+ and CD8+ T cells in HIV mono-infection and co-infection with LTBI or TB. **(D).** We observed significant difference between the polyfunctionality profiles of HIV+/LTBI and HIV+/TB groups for both CD4+ (p = 0.007) and CD8+ (p = 0.19). A highly polyfunctional MTB-specific CD4+ T cell cytokine profile was observed in HIV positive subjects co-infected with LTBI, which including the capacity to secrete four cytokines by 12% of MTB-specific T cells (IFN^+^IL-2^+^IL-17^+^TNFα (9%), or IFN^+^IL-21^+^IL-17^+^TNFα (3%)). **(E)** A decrease in the polyfunctional profile of MTB-specific CD4+ T cells was observed in HIV co-infection with TB, with an increased dominance in mono-functional TNFα producing cells (from 12% to 31%). **(F)** MTB-specific CD8+ T cells from HIV infected subjects co-infected with LTBI displayed a profile with a maximum of 2 functions being present (IFN^+^TNFα (41%)). **(G)** Mono-functional IFNγ and TNFα producing cells dominated the profile of MTB-specific CD8+ cells in HIV infected subjects co-infected with TB (at 78%). There was total loss of IL-2 function but IFNγ^+^TNF^+^ double positive cells were present (IFN^+^TNFα^+^ (22%)). An increased dominance in mono-functional TNFα producing cells was also observed (47%).

Interestingly, when we assessed MTB-specific T cells functionality, we observed a decline in functionality (both in amount and number of cytokines secreted) in HIV-TB co-infected individuals compared to those with HIV-LTBI (Fig. 2). Mono-functional IFN-γ and TNF-α producing cells dominated the profile of MTB-specific CD8+ cells in HIV infected subjects co-infected with TB (at 78%) (Fig. 2I).

**Fig 3:**
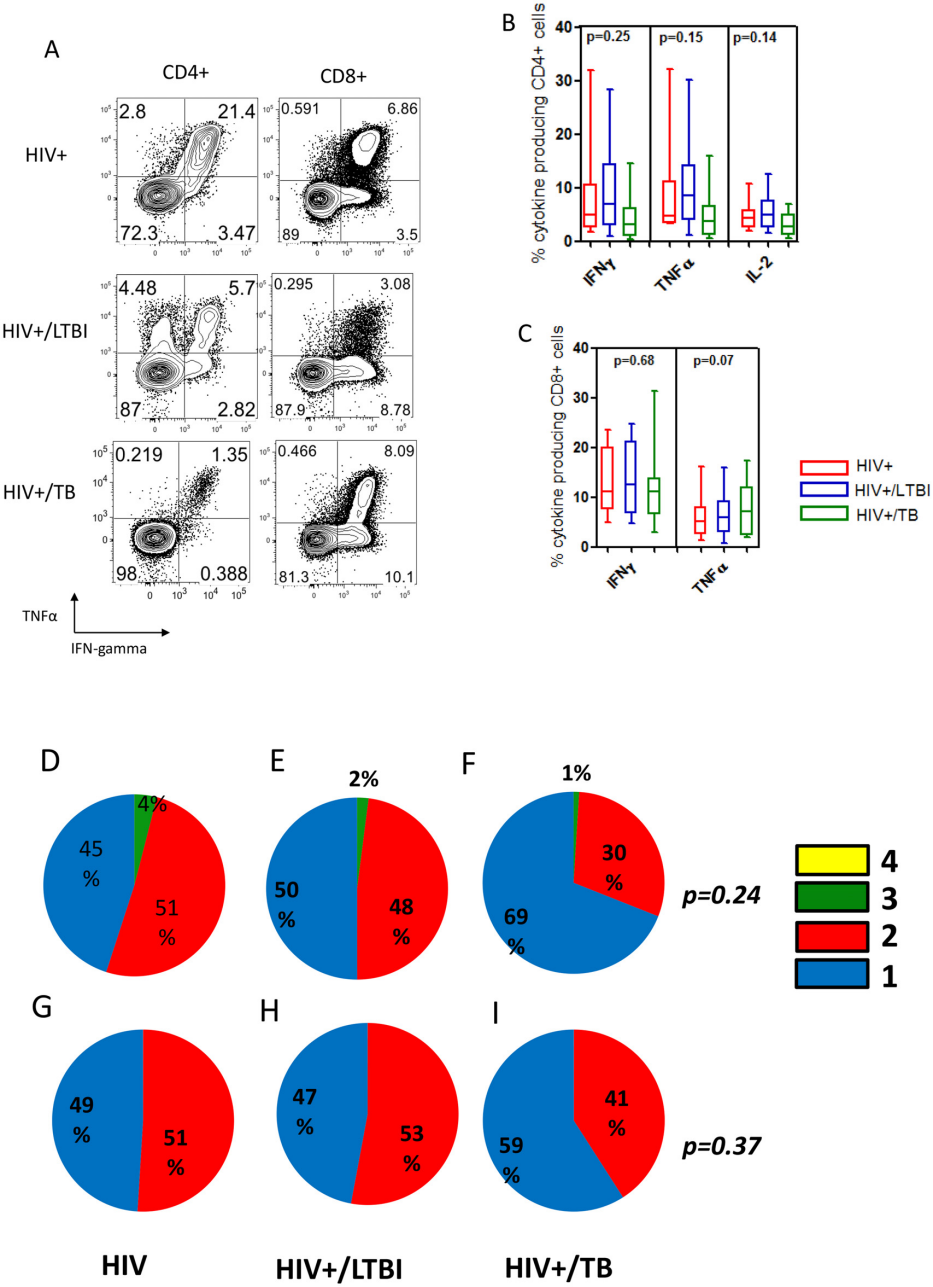
Non-specific CD4+ and CD8+ T cell functionality is reduced in HIV infected individuals co-infected with latent Mycobacterium tuberculosis infection (LTBI) and active tuberculosis (TB) disease. We assessed non-specific cytokine production following 6 hour SEB stimulation. **(A)** Representative flow cytometry plots showing cytokine responses for SEB stimulated CD4 and CD8 T cells from all patient categories. **(B, C)** No quantitative differences were observed when non-specific cytokine production was compared amongst patient groups. Whilst no significant differences were observed, HIV/TB co-infected subjects appeared to produce less cytokine in response to SEB. **(D, E, F)** Analysis of non-specific CD4 polyfunctionality revealed an increase (p = 0.007) in mono-functional cells from HIV+ mono-infection to HIV/LTBI to HIV/TB. Analysis of non-specific CD8+ T cell polyfunctionality showed no significant difference between the groups (p = 0.19) **(G, H, I)**.

We additionally assessed non-specific cytokine production following SEB stimulation (Fig. 3A-I). Whilst no significant quantitative differences were observed, HIV/TB co-infected subjects appeared to produce less cytokine in response to SEB as compared to HIV/LTBI and HIV mono-infected subjects. Analysis of non-specific CD4+ T cell polyfunctionality revealed a higher quantity in mono-functional cells from HIV mono-infection (Fig. 3D) to HIV/LTBI (Fig. 3E) to HIV+/TB (Fig. 3F). Analysis of non-specific CD8+ T cell polyfunctionality indicated no significant changes between the groups (Fig. 3G-I).

## Discussion

HIV-specific CD4+ and CD8+ T cell functionality was found to be lower in co-infection with LTBI, and to a greater extent TB, as compared to HIV mono-infection. The observed changes in HIV-specific T cell single cytokine release may not necessarily be a loss in functional capacity but rather a loss of HIV-specific T cells. However, the polyfunctionality data are rather striking, showing that MTB co-infection resulted in a reduced ability of HIV-specific T cells to co-secrete multiple cytokines, suggesting that MTB infection augments HIV- specific T cell dysregulation.

In HIV mono-infection, several studies have highlighted the relationship between high-levels of T cell polyfunctionality and the control of HIV disease progression, with increased viral load being linked to a decrease in HIV-specific T cell function [8],[12]. HIV-specific CD4+ T cells in our HIV mono-infected subjects secreted IFN-y, IL-2, TNF-α, IL-21 and IL-17. The loss of each of these functions individually could have a significant impact on HIV control. For example, IL-21 has been strongly associated with mechanisms of viral control in elite controllers [13]. HIV-specific CD8+ T cells in our HIV mono-infected subjects were shown to secrete predominantly TNF-α and IFN-γ withextremely low amounts of IL-2[8]. A loss in the ability of CD8+ T cells to produce IL-2 has been shown to be associated with T cell exhaustion [8].

For both HIV-specific CD4+ and CD8+ T cells we observed a decline in polyfunctional capacity in MTB co-infection suggesting that mycobacterial load may a role.This is supported by lower MTB-specific T cell functionality in TB co-infection with HIV compared to LTBI. Day *et al*, (2011) have previously demonstrated that the functional capacity (specifically IL-2, TNF-α and IFNγ) of MTB-specific CD4+ T cells is decreased in TB compared to LTBI mono-infection. Our data extend these findings by additionally assessing IL-21 and IL-17 secretion capacity in HIV and MTB co-infection. Our data suggest that the loss of T cell function associated with TB may extend beyond HIV and MTB-specific responses, as our preliminary data reveals impairment in SEB-specific responses in this co-infection state. The high levels of inflammation and immune activation present in TB may enhance general immune exhaustion and T cell anergy [14].

Importantly, cytokine T cell profiles may be able to act as biomarkers of specific disease states. Mono-functional TNF-α producing cells dominated the MTB-specific T cell polyfunctionality profiles in those co-infected with TB as compared to those co-infected with LTBI. This confirms and extends the predictive model by Harari *et al* which showed that TNF-α single positive CD4+ T cells can differentiate between LTBI and TB in MTB mono-infected individuals [9]. More importantly, we show that this may hold true in HIV co-infection where diagnosing TB is problematic using currently available assays. Mono-functional TNF-α secreting T cells may therefore be an effective diagnostic biomarker for active TB in HIV positive populations. Interestingly, TNF-α generated in response to MTB infection has been shown to increase HIV viral replication suggesting that the cytokine profiles and dominance of TNF-α single producing T cells in TB, may contribute to increased HIV replication and disease progression[15]. In addition, our data suggests that the loss of T cell function associated with TB may extend beyond HIV and MTB-specific responses, as our preliminary data revealed impairment in SEB-specific responses in this co-infection state. The high levels of inflammation and immune activation present in TB may enhance general immune exhaustion and T cell anergy [14].

In conclusion, our results indicate that MTB infection is linked to lower HIV-specific CD8+ and CD4+ T cell polyfunctionality. As HIV-specific T cells are most defective in HIV co-infection with TB, as compared to LTBI, mycobacterial load may contribute to the loss of T cell function. Decreased T cell function may be a contributing factor to increased HIV disease progression in co-infection as functionally defective HIV-specific T cells may be less able to control HIV.
