# Correction: Co-Infection with *Mycobacterium tuberculosis* Impairs HIV-Specific CD8+ and CD4+ T Cell Functionality

**DOI:** 10.1371/journal.pone.0127763

**Published:** 2015-05-20

**Authors:** The PLOS ONE Staff

## Notice of Republication

This article was republished on May 5, 2015, to correct Figs 1–3, which were missing several panels. Please download this article again to view the correct version. The originally published, uncorrected article and the republished, corrected article are provided here for reference.

## Supporting Information

S1 File Originally published, uncorrected article. (PDF)

S2 File Republished, corrected article. (PDF)
