# Supplementary material for: Correction: Co-Infection with Mycobacterium tuberculosis Impairs HIV-Specific CD8+ and CD4+ T Cell Functionality
Source: PLoS One. 2015 May 20;10(5):e0127763. doi: 10.1371/journal.pone.0127763 (PMC4439175; doi:10.1371/journal.pone.0127763)
Supplement: S1 File — (PDF) [file pone.0127763.s001.pdf]

PLOS | ONE

RESEARCH ARTICLE

Co-Infection with *Mycobacterium tuberculosis* Impairs HIV-Specific CD8+ and CD4+ T Cell Functionality

Shivan Chetty[1,2], Pamla Govender[1], Jennifer Zupkosky[3], Mona Pillay[1], Musie Ghebremichael[3], Mahomed-Yunus S. Moosa[4], Thumbi Ndung'u[1,2,3,5], Filippos Porichis[3☯], Victoria O. Kasprowicz[1,2,3☯]*

1 HIV Pathogenesis Programme, Doris Duke Medical Research Institute, University of KwaZulu-Natal, Durban, South Africa, **2** KwaZulu-Natal Research Institute for Tuberculosis and HIV (K-RITH), University of KwaZulu-Natal, Durban, South Africa, **3** The Ragon Institute of MGH, MIT and Harvard, Cambridge, Massachusetts, United States of America, **4** Department of Infectious Disease, Division of Internal Medicine, Nelson R. Mandela School of Medicine, University of KwaZulu-Natal, Durban, South Africa, **5** Max Planck Institute for Infection Biology, Berlin, Germany

☯ These authors contributed equally to this work.
* vkasprowicz@mgh.harvard.edu

OPEN ACCESS

Citation: Chetty S, Govender P, Zupkosky J, Pillay M, Ghebremichael M, Moosa M-YS, et al. (2015) Co-Infection with *Mycobacterium tuberculosis* Impairs HIV-Specific CD8+ and CD4+ T Cell Functionality. PLoS ONE 10(3): e0118654. doi:10.1371/journal.pone.0118654

Academic Editor: William A Paxton, Institute of Infection and Global Health, UNITED KINGDOM

Received: July 23, 2014

Accepted: January 9, 2015

Published: March 17, 2015

Copyright: © 2015 Chetty et al. This is an open access article distributed under the terms of the Creative Commons Attribution License, which permits unrestricted use, distribution, and reproduction in any medium, provided the original author and source are credited.

Data Availability Statement: All relevant data are within the paper.

Funding: The authors would like to thank the following funders: the Howard Hughes Medical Institute (support for T. N., V. O. K.),the Harvard University CFAR grant (P30 AI060354), the South African Medical Research Council (MRC) and The CU-SA Fogarty AITRP programme (S. C.), the National Heart Lung and Blood Institute of the National Institutes of Health (RO1 HL-092565 and R37 AI067073 and P01 AI-080192). F. P. is supported by fellowship grants of the Massachusetts General

Abstract

The ability of antigen-specific T cells to simultaneously produce multiple cytokines is thought to correlate with the functional capacity and efficacy of T cells. These 'polyfunctional' T cells have been associated with control of HIV. We aimed to assess the impact of co-infection with *Mycobacterium tuberculosis* (MTB) on HIV-specific CD8+ and CD4+ T cell function. We assessed T cell functionality in 34 South African adults by investigating the IFN-y, IL-2, TNF-α, IL-21 and IL-17 cytokine secretion capacity, using polychromatic flow cytometry, following HIV Gag-specific stimulation of peripheral blood mononuclear cells. We show that MTB is associated with lower HIV-specific T cell function in co-infected as compared to HIV mono-infected individuals. This decline in function was greatest in co-infection with active Tuberculosis (TB) compared to co-infection with latent MTB (LTBI), suggesting that mycobacterial load may contribute to this loss of function. The described impact of MTB on HIV-specific T cell function may be a mechanism for increased HIV disease progression in co-infected subjects as functionally impaired T cells may be less able to control HIV.

Introduction

HIV and Tuberculosis (TB) are severe global dual-epidemics. Data suggest that co-infection with HIV and *Mycobacterium tuberculosis* (MTB) increases disease progression of both diseases[1]. For example, higher HIV viral loads are observed in MTB co-infection and increased HIV replication occurs in MTB infected macrophages [2, 3]. The high levels of inflammation and immune activation, as present in TB, may create an optimal cytokine milieu for HIV

Hospital Executive Committee on Research and the Harvard Global Health Institute (HGHI). T. N. is also supported by the South African Research Chairs Initiative and the Victor Daitz Foundation. Open access publication of this article has been made possible through support from the Victor Daitz Information Gateway, an initiative of the Victor Daitz Foundation and the University of KwaZulu-Natal. The funders had no role in study design, data collection and analysis, decision to publish, or preparation of the manuscript.

Competing Interests: The authors have declared that no competing interests exist.

replication[4]. Whilst immunological impairment is likely to contribute to the increased morbidity and mortality associated with co-infection, the specific mechanisms remain largely unknown. Several studies have reported an impact of HIV on MTB-specific T cell immunity [5,6,7]. For example, increased infection and lysis of MTB-specific T cells has been accredited to HIV infection [5, 6]. Day *et al* showed that HIV infection impairs MTB-specific responses in HIV co-infection with LTBI, demonstrating that the proportion of IL-2 secreting MTB-specific CD4+ T cells inversely correlated with HIV viral load [7].

The ability of antigen-specific T cells to simultaneously produce multiple cytokines is believed to correlate with the functional capacity and efficacy of T cells. Frequency of these 'polyfunctional' T cells in blood samples from infected subjects has been associated with clinical control of HIV and TB [8, 9]. For example, higher bacterial load has been shown to decrease MTB-specific T cell functionality and mono-functional T cells have been shown to dominate functionality profiles in TB as compared to LTBI [10]. Harari *et al* have reported that greater proportions of TNF-α single-positive CD4 T cells are present in individuals with active TB as compared with LTBI [9]. If and how MTB co-infection affects HIV-specific T cell function and polyfunctionality is unknown.

Methods

Participants and Study Samples

We enrolled 13 HIV positive individuals with active TB, 9 HIV positive individuals with latent MTB (LTBI), and 11 HIV positive individuals without evidence of LTBI or active TB (Table 1).

Table 1. Viral load and CD4 count information for study participants.

HIV+				HIV+/LTBI				HIV+/TB			
Patient ID.	CD4 Count (cells/ul)	Viral Load	Sex	Patient ID.	CD4 Count (cells/ul)	Viral Load	Sex	Patient ID.	CD4+ count (cells/ul)	Viral Load	Sex
SK 010 B21	218	5822	F	SK 141 B20	278	2676	F	PID 1079	209	2900	M
SK 114 B14	274	29100	F	SK 195 B20	207	10077	M	PID 1120	460	3700	M
SK 139 B20	184	47547	F	SK 236 B9	184	272227	F	PID 1135	253	470000	M
SK 142 B12	275	186858	F	SK 351 B15	350	285891	F	PID 1224	345	3850	M
SK 208 B11	126	25523	F	SK 359 B15	466	9449	F	PID 1225	366	224137	M
SK 212 B22	233	19670	F	SK 364 B15	285	3140	M	PID 821	110	20000	F
SK 278 B17	443	26036	F	SK 391 B13	247	1936	F	PID 683	195	430000	F
SK 324 B14	353	144252	F	SK 397 B14	332	198266	M	PID 863	204	49000	M
SK 373 B15	184	58237	F	SK 425 B13	192	101600	F	PID 917	239	66000	F
SK 410 B11	495	2570	F					PID 929	270	540000	M
SK 444 B13	331	21447	F					PID1024	239	88000	M
								PID1046	267	17000	M

doi:10.1371/journal.pone.0118654.t001

All were chronically infected HIV positive South-African adults and were CD4 T cell count matched. Viral loads did not significantly differ between patient groups (p = 0.978). TB was identified by a positive sputum acid-fast bacillus smear or sputum culture. LTBI was defined as a positive ESAT-6/CFP-10 IFN-gamma ELISPOT, in the absence of signs and symptoms of TB [11]. Ethical approval and written informed consent from participants was obtained (University of KwaZulu-Natal Biomedical Research Ethics Committee: E028/99 and H020/06). Patients were anti-retroviral treatment naive and not receiving anti-TB treatment.

Flow cytometry

We assessed T cell functionality using a multi-parameter flow cytometry panel: Viability marker, CD3, CD4, CD8, IFNγ, IL-2, TNF-α, IL-21 and IL-17. Intracellular cytokine staining (ICS) of peripheral blood mononuclear cells (PBMC) was performed following a 6 hour stimulation with either Staphylococcal enterotoxin B (SEB), an HIV Gag peptide pool, or an MTB-specific ESAT-6/CFP-10 peptide pool. FlowJo (version 8.3.3; Treestar) and GraphPad Prism (V.5.5) software were used to analyze the data. A positive antigen-specific response was defined as greater than or equal to 0.05% of the T cell subset analyzed, and 3 times above background.

Statistical analysis

GraphPad Prism (V.5.5) was used to perform all statistical analysis. Mann-Whitney test was used to compare continuous outcomes between two groups. For more than two groups comparison, Kruskall-Wallis test with Dunn's post hoc analyses was used. F Fisher's exact test was used to compare categorical outcomes (i.e., pie charts). All *p* values are two sided and a p-value$<$0.05 was considered significant.

Results

HIV-specific CD4 T cells were readily detectable in mono-infected individuals (Fig. 1A and 1B). HIV-specific CD4+ cell release of IFN-γ was significantly lower in HIV+/TB as compared to HIV+/LTBI (p = 0.005)(Fig. 1B). HIV-specific CD4+ cell release of TNF-α (p = 0.01) and IL-2 (p$<$0.001) were significantly lower in HIV+/TB as compared to HIV mono-infected individuals. In summary, HIV co-infection with MTB was associated with a decrease in the amount of cytokine (IFN-γ, TNF-α and IL-2) secreted for HIV-specific CD4+ T cells.

HIV-specific CD8+ release of IFN-γ (p$<$0.001) and TNFα (p = 0.004) were significantly lower in HIV/TB co-infected subjects as compared to HIV mono-infected subjects (Fig. 1C). Additionally, IFN-γ secretion was found to be significantly lower in the HIV+/TB group as compared to the HIV+/LTBI group (p = 0.02). No significant antigen-specific production of IL-2, IL-17 or IL-21 was observed for CD8+ T cell responses. In summary, HIV co-infection with MTB was associated with a decrease in the amount of cytokine (TNF-α and IFN-γ) secreted for HIV-specific CD8+ T cells.

Significant differences were also observed between the polyfunctionality profiles for CD4+ (p = 0.04) and CD8+ (p$<$0.001) T cells (Fig. 1 D-I). HIV mono-infected subjects displayed a polyfunctional HIV-specific CD4+ T cell profile with a maximum of four functions (IFN-γ^+IL-2^+IL-17^+TNF-α^+ (2% of HIV-specific CD4+ T cells)(Fig. 1d). 15% of HIV-specific CD4 T cells were able to secrete 3 cytokines simultaneously (IFN-γ^+IL-2^+TNF-α^+), 26% secreted 2 cytokines (IFN-γ^+TNF-α^+(15%),IFN-γ^+IL-2^+(6%), IL-2^+IL-21^+(5%))while 57% were mono-functional (IFN-γ^+(31%), TNF-α^+(26%)). HIV-specific four-function CD4+ T cells were not present in those co-infected with LTBI and this was accompanied by a higher proportion of mono-functional T cells: 65%(IFN-γ^+(29%),TNF-α^+(36%))(Fig. 1E).Further changes in HIV-specific CD4+ T cell polyfunctionality were observed in HIV positive subjects co-infected with

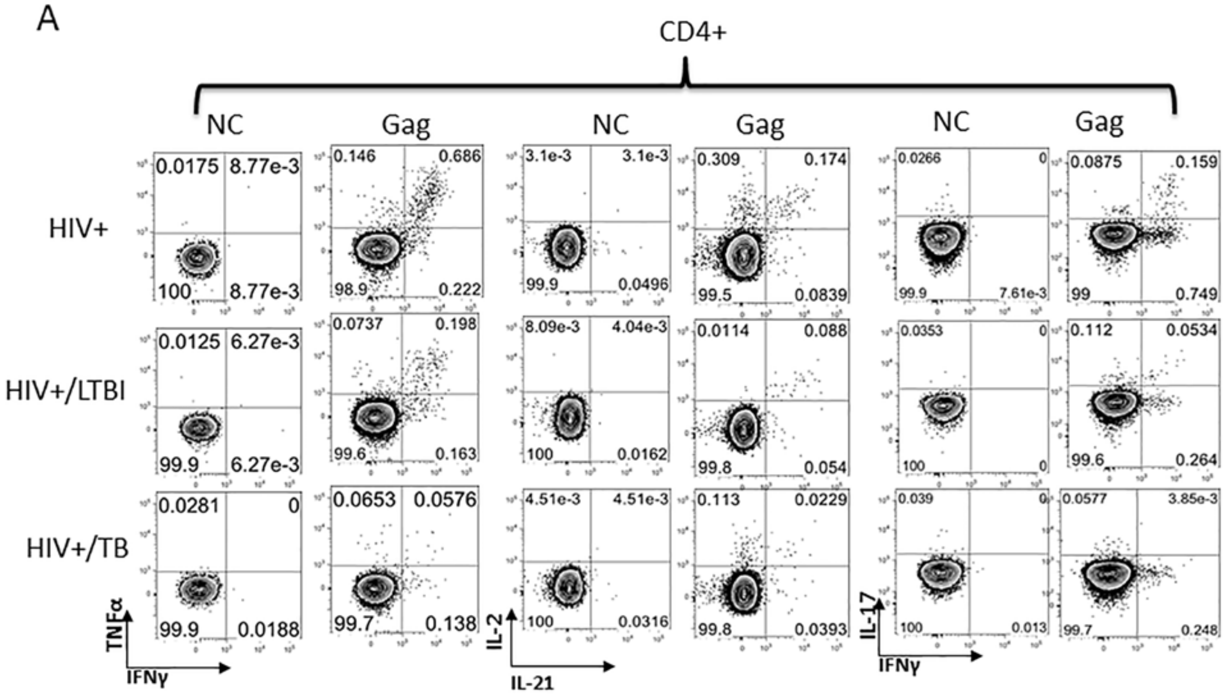

Fig 1. HIV specific CD4+ and CD8+ T cell functionality is reduced in HIV infected individuals co-infected with latent Mycobacterium tuberculosis infection (LTBI) and active Tuberculosis (TB) disease. (A) Representative flow cytometry plots showing cytokine responses for HIV (gag), control (no antigen) for HIV mono-infected subjects, and subjects co-infected with latent MTB infection (LTBI) and active tuberculosis (TB) **(B)** HIV-specific CD4+ release of IFNγ ($p<0.001$), TNFα ($p<0.01$) and IL-2 ($p<0.01$) were significantly different between all groups (Kruskal-Wallis). **(C)** HIV-specific CD8+ release of IFNγ ($p<0.01$) and TNFα ($p<0.001$) was significantly lower from HIV mono-infected subjects, to those co-infected with active TB. We additionally assessed the polyfunctionality profile of HIV specific CD4+ and CD8+ T cells in all patient groups. The polyfunctionality profiles between groups differed significantly for both CD4+ ($p = 0.04$) and CD8+ ($p<0.001$).**(D)** HIV-specific CD4+ T cells from HIV mono-infected subjects displayed a polyfunctional CD4+ T cell profile with a maximum of four functions being present ($IFN^{+}IL\text{-}2^{+}IL\text{-}17^{+}TNF\alpha$ (2%)).**(E)** HIV-specific four function CD4+ T cells were not present in subjects co-infected with LTBI **(F)** Further decreases in HIV-specific CD4+ T cell polyfunctionality were observed in HIV positive subjects co-infected with TB, being replaced by a largely mono-functional profile with a decreased amount of triple cytokine cells (5% as compared to 13% in HIV/LTBI and 15% in HIV mono-infection). Additionally, single positive TNF-α cells dominated the profile (48%). **(G)** HIV-specific CD8+ T cells in HIV mono-infected subjects displayed a polyfunctional profile with a maximum of 2 functions being present (IFN^{+} TNFα (2%). **(H)** A maximum of 2 functions ($IFN^{+}TNF\alpha^{+}$ (28%)) were present in HIV-specific CD8+ cells from subjects co-infected with LTBI. **(I)** 87% mono-functional cells were present in the HIV-specific CD8+T cell profile from subjects co-infected with TB, suggesting a complete loss of polyfunctionality.

doi:10.1371/journal.pone.0118654.g001

TB, with only 5% being able to co-secrete 3 cytokines ($IFN\text{-}\gamma^{+}IL\text{-}2^{+}TNF\text{-}\alpha^{+}$) and 76% with mono-functional secretion capacity (TNF-α only (47%), IL-2 only (2%), or IFN-γ only (26%)) (Fig. 1G). In summary, HIV co-infection with MTB was associated with a decrease in the number of cytokines secreted from HIV-specific CD4+ T cells.

In HIV mono-infected individuals the HIV-specific CD8+ T cell profile consisted of dual cytokine secreting cells $IFN\text{-}\gamma^{+}TNF\text{-}\alpha^{+}$ (38% of total HIV-specific CD8+ T cells) and mono-functional cells (62% of total HIV-specific CD8+ T cells)(Fig. 1G).Bi-functional T cells were found to be present at progressively lower frequencies from HIV mono-infection, to co-infection with LTBI, to co-infection with TB (Fig. 1 G-1). 87% mono-functional HIV-specific CD8+ T cells were present in subjects co-infected with TB (Fig. 1I). In summary, HIV co-infection with MTB was associated with a decrease in the number of cytokines secreted from HIV-specific CD8+ T cells.

Interestingly, when we assessed MTB-specific T cells functionality, we observed a decline in functionality (both in amount and number of cytokines secreted) in HIV-TB co-infected individuals compared to those with HIV-LTBI (Fig. 2). Mono-functional IFN-γ and TNF-α

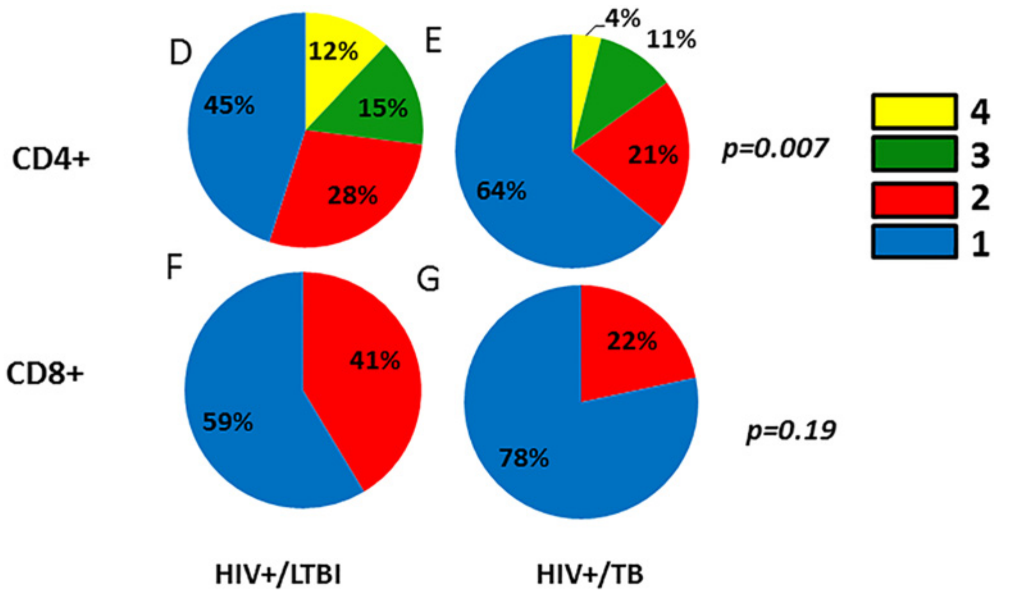

Fig 2. MTB-specific CD4+ and CD8+ T cell functionality is reduced in HIV infected individuals co-infected with latent Mycobacterium tuberculosis infection (LTBI) and active tuberculosis (TB) disease. (A) Representative flow cytometry plots showing cytokine IFN-gamma and TNF-α response for Mycobacterium tuberculosis (MTB) (CFP-10 and ESAT-6). Corresponding negative controls (no antigen) are the same as shown in Fig. 1B. **(B)** MTB-specific CD4+ release of IFNγ (p = 0.03), TNFα (p<0.03) and IL-21 (p<0.002) were observed to be significantly lower from subjects co-infected with LTBI to those co-infected with TB. **(C)** MTB-specific CD8+ release of IFNγ (p = 0.022) and TNFα (p<0.011), were lower subjects co-infected with TB as compared co-infected with LTBI. We next assessed the polyfunctionality profile of Mycobacterium tuberculosis (MTB)—specific CD4+ and CD8+ T cells in HIV mono-infection and co-infection with LTBI or TB. **(D).** We observed significant difference between the polyfunctionality profiles of HIV+/LTBI and HIV+/TB groups for both CD4+ (p = 0.007) and CD8+ (p = 0.19). A highly polyfunctional MTB-specific CD4+ T cell cytokine profile was observed in HIV positive subjects co-infected with LTBI, which including the capacity to secrete four cytokines by 12% of MTB-specific T cells ($IFN^{+}IL\text{-}2^{+}IL\text{-}17^{+}TNF\alpha$ (9%), or $IFN^{+}IL\text{-}21^{+}IL\text{-}17^{+}TNF\alpha$ (3%)). **(E)** A decrease in the polyfunctional profile of MTB-specific CD4+ T cells was observed in HIV co-infection with TB, with an increased dominance in mono-functional TNFα producing cells (from 12% to 31%). **(F)** MTB-specific CD8+ T cells from HIV infected subjects co-infected with LTBI displayed a profile with a maximum of 2 functions being present ($IFN^{+}TNF\alpha$ (41%)). **(G)** Mono-functional IFNγ and TNFα producing cells dominated the profile of MTB-specific CD8+ cells in HIV infected subjects co-infected with TB (at 78%). There was total loss of IL-2 function but $IFN\gamma^{+}TNF^{+}$ double positive cells were present ($IFN^{+}TNF\alpha^{+}$ (22%)). An increased dominance in mono-functional TNFα producing cells was also observed (47%).

doi:10.1371/journal.pone.0118654.g002

producing cells dominated the profile of MTB-specific CD8+ cells in HIV infected subjects co-infected with TB (at 78%) (Fig. 2I).

We additionally assessed non-specific cytokine production following SEB stimulation (Fig. 3A-I). Whilst no significant quantitative differences were observed, HIV/TB co-infected subjects appeared to produce less cytokine in response to SEB as compared to HIV/LTBI and HIV mono-infected subjects. Analysis of non-specific CD4+ T cell polyfunctionality revealed a higher quantity in mono-functional cells from HIV mono-infection (Fig. 3D) to HIV/LTBI (Fig. 3E) to HIV+/TB (Fig. 3F). Analysis of non-specific CD8+ T cell polyfunctionality indicated no significant changes between the groups (Fig. 3G-I).

Discussion

HIV-specific CD4+ and CD8+ T cell functionality was found to be lower in co-infection with LTBI, and to a greater extent TB, as compared to HIV mono-infection. The observed changes in HIV-specific T cell single cytokine release may not necessarily be a loss in functional

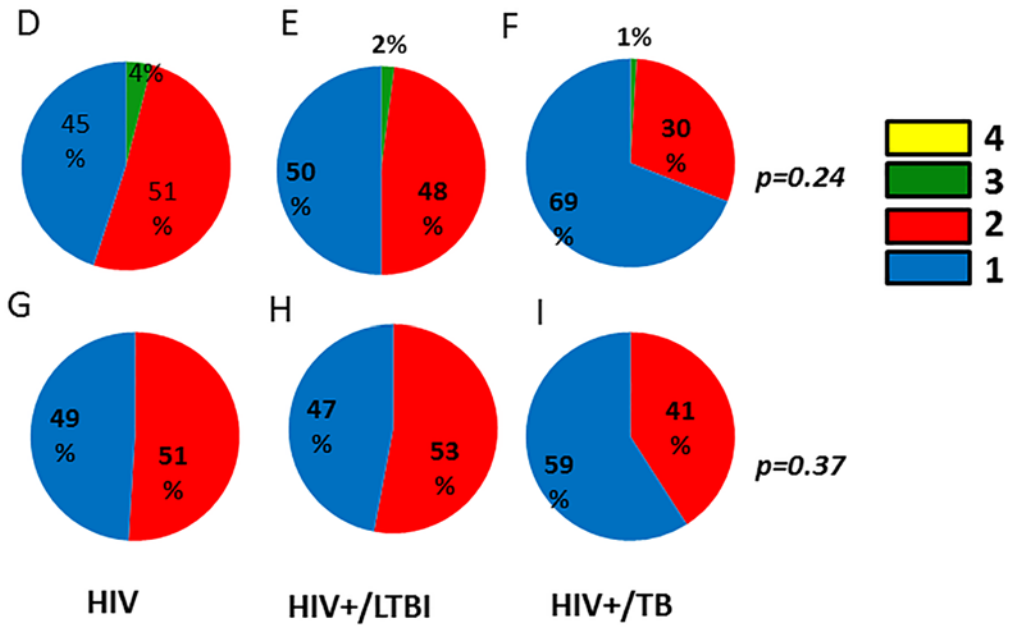

Fig 3. Non-specific CD4+ and CD8+ T cell functionality is reduced in HIV infected individuals co-infected with latent Mycobacterium tuberculosis infection (LTBI) and active tuberculosis (TB) disease. We assessed non-specific cytokine production following 6 hour SEB stimulation. **(A)** Representative flow cytometry plots showing cytokine responses for SEB stimulated CD4 and CD8 T cells from all patient categories. **(B, C)** No quantitative differences were observed when non-specific cytokine production was compared amongst patient groups. Whilst no significant differences were observed, HIV/TB co-infected subjects appeared to produce less cytokine in response to SEB. **(D, E, F)** Analysis of non-specific CD4 polyfunctionality revealed an increase (p = 0.007) in mono-functional cells from HIV+ mono-infection to HIV/LTBI to HIV/TB. Analysis of non-specific CD8+ T cell polyfunctionality showed no significant difference between the groups (p = 0.19) **(G, H, I)**.

doi:10.1371/journal.pone.0118654.g003

capacity but rather a loss of HIV-specific T cells. However, the polyfunctionality data are rather striking, showing that MTB co-infection resulted in a reduced ability of HIV-specific T cells to co-secrete multiple cytokines, suggesting that MTB infection augments HIV- specific T cell dysregulation.

In HIV mono-infection, several studies have highlighted the relationship between high-levels of T cell polyfunctionality and the control of HIV disease progression, with increased viral load being linked to a decrease in HIV-specific T cell function [8],[12]. HIV-specific CD4+ T cells in our HIV mono-infected subjects secreted IFN-y, IL-2, TNF-α, IL-21 and IL-17. The loss of each of these functions individually could have a significant impact on HIV control. For example, IL-21 has been strongly associated with mechanisms of viral control in elite controllers [13]. HIV-specific CD8+ T cells in our HIV mono-infected subjects were shown to secrete predominantly TNF-α and IFN-γ withextremely low amounts of IL-2[8]. A loss in the ability of CD8+ T cells to produce IL-2 has been shown to be associated with T cell exhaustion [8].

For both HIV-specific CD4+ and CD8+ T cells we observed a decline in polyfunctional capacity in MTB co-infection suggesting that mycobacterial load may a role.This is supported by lower MTB-specific T cell functionality in TB co-infection with HIV compared to LTBI. Day *et al*, (2011) have previously demonstrated that the functional capacity (specifically IL-2, TNF-α and IFNγ) of MTB-specific CD4+ T cells is decreased in TB compared to LTBI mono-infection. Our data extend these findings by additionally assessing IL-21 and IL-17 secretion capacity in HIV and MTB co-infection. Our data suggest that the loss of T cell function associated with TB may

extend beyond HIV and MTB-specific responses, as our preliminary data reveals impairment in SEB-specific responses in this co-infection state. The high levels of inflammation and immune activation present in TB may enhance general immune exhaustion and T cell anergy [14].

Importantly, cytokine T cell profiles may be able to act as biomarkers of specific disease states. Mono-functional TNF-α producing cells dominated the MTB-specific T cell polyfunctionality profiles in those co-infected with TB as compared to those co-infected with LTBI. This confirms and extends the predictive model by Harari *et al* which showed that TNF-α single positive CD4+ T cells can differentiate between LTBI and TB in MTB mono-infected individuals [9]. More importantly, we show that this may hold true in HIV co-infection where diagnosing TB is problematic using currently available assays. Mono-functional TNF-α secreting T cells may therefore be an effective diagnostic biomarker for active TB in HIV positive populations. Interestingly, TNF-α generated in response to MTB infection has been shown to increase HIV viral replication suggesting that the cytokine profiles and dominance of TNF-α single producing T cells in TB, may contribute to increased HIV replication and disease progression[15]. In addition, our data suggests that the loss of T cell function associated with TB may extend beyond HIV and MTB-specific responses, as our preliminary data revealed impairment in SEB-specific responses in this co-infection state. The high levels of inflammation and immune activation present in TB may enhance general immune exhaustion and T cell anergy [14].

In conclusion, our results indicate that MTB infection is linked to lower HIV-specific CD8+ and CD4+ T cell polyfunctionality. As HIV-specific T cells are most defective in HIV co-infection with TB, as compared to LTBI, mycobacterial load may contribute to the loss of T cell function. Decreased T cell function may be a contributing factor to increased HIV disease progression in co-infection as functionally defective HIV-specific T cells may be less able to control HIV.

Acknowledgments

We would like to thank Sr Kesia Ngwenya and the staff and participants of the Sinikithimba Cohort at McCord Hospital as well as Dr Faieza Sahid and the String Study clinical team at King Edward Hospital. We also thank Daniel Kaufmann and Bruce Walker for helpful discussions. We would like to thank the following funders: the Howard Hughes Medical Institute (support for T.N., V.O.K),the Harvard University CFAR grant (P30 AI060354), the South African Medical Research Council (MRC) and The CU-SA Fogarty AITRP programme (S.C), the National Heart Lung and Blood Institute of the National Institutes of Health (RO1 HL-092565 and R37 AI067073 and P01 AI-080192). F.P. is supported by fellowship grants of the Massachusetts General Hospital Executive Committee on Research and the Harvard Global Health Institute (HGHI). T.N. is also supported by the South African Research Chairs Initiative and the Victor Daitz Foundation. Open access publication of this article has been made possible through support from the Victor Daitz Information Gateway, an initiative of the Victor Daitz Foundation and the University of KwaZulu-Natal. The funders had no role in study design, data collection and analysis, decision to publish, or preparation of the manuscript.

Author Contributions

Conceived and designed the experiments: FP VOK TN MYM. Performed the experiments: SC PG JZ MP. Analyzed the data: SC FP VOK MG. Contributed reagents/materials/analysis tools: VOK MYM. Wrote the paper: SC FP VOK MYM.

References

1. Karim SSA, Churchyard GJ, Karim QA, Lawn SD. HIV infection and tuberculosis in South Africa: an urgent need to escalate the public health response. The Lancet 2009; 374:921–933 doi: 10.1016/S0140-6736(09)60916-8 PMID: 19709731
2. Goletti D, Weissman D, Jackson RW, Collins F, Kinter A, Fauci AS. The in vitro induction of human immunodeficiency virus (HIV) replication in purified protein derivative-positive HIV-infected persons by recall antigen response to Mycobacterium tuberculosis is the result of a balance of the effects of endogenous interleukin-2 and proinflammatory and antiinflammatory cytokines. J Infect Dis 1998; 177:1332–8 PMID: 9593021
3. Hoshino Y, Nakata K, Hoshino S, Honda Y, Tse D, Shioda T, et al. Maximal HIV-1 replication in alveolar macrophages during tuberculosis requires both lymphocyte contact and cytokines. J Exp Med 2002; 195:495–505 PMID: 11854362
4. Stacey AR, Norris PJ, Qin L, Haygreen E, Taylor E, Heitman J, et al. Induction of a Striking Systemic Cytokine Cascade prior to Peak Viremia in Acute Human Immunodeficiency Virus Type 1 Infection, in Contrast to More Modest and Delayed Responses in Acute Hepatitis B and C Virus Infections. J Virol 2009; 83:3719–3733 doi: 10.1128/JVI.01844-08 PMID: 19176632
5. Geldmacher C, Schuetz A, Ngwenyama N, Casazza J, Sanga E, Saathoff E, et al. Early depletion of Mycobacterium tuberculosis-specific T helper 1 cell responses after HIV-1 infection. J Infect Dis 2008; 198:1590–8 doi: 10.1086/593017 PMID: 19000013
6. Geldmacher C, Ngwenyama N, Schuetz A, Petrovas C, Reither K, Heeregrave E, et al. Preferential infection and depletion of Mycobacterium tuberculosis—specific CD4 T cells after HIV-1 infection. J Exp Med 2010; 207:2869–2881 doi: 10.1084/jem.20100090 PMID: 21115690
7. Day CL, Mkhwanazi N, Reddy S, Mncube Z, van der Stok M, Klenerman P, et al. Detection of polyfunctional Mycobacterium tuberculosis-specific T cells and association with viral load in HIV-1-infected persons. J Infect Dis 2008; 197:990–9 doi: 10.1086/529048 PMID: 18419535
8. Betts MR, Nason MC, West SM, De Rosa SC, Migueles SA, Abraham J, et al. HIV nonprogressors preferentially maintain highly functional HIV-specific CD8+ T cells. Blood 2006; 107:4781–9 PMID: 16467198
9. Harari A, Rozot V, Enders FB, Perreau M, Stalder JM, Nicod LP, et al. Dominant TNF-alpha+ Mycobacterium tuberculosis-specific CD4+ T cell responses discriminate between latent infection and active disease. Nat Med 2011; 17:372–6 doi: 10.1038/nm.2299 PMID: 21336285
10. Day CL, Abrahams DA, Lerumo L, Janse van Rensburg E, Stone L, O'rie T, et al. Functional capacity of Mycobacterium tuberculosis-specific T cell responses in humans is associated with mycobacterial load. J Immunol 2011; 187:2222–32 doi: 10.4049/jimmunol.1101122 PMID: 21775682
11. Mitchell JE, Chetty S, Govender P, Pillay M, Jaggernath M, Kasmar A, et al. Prospective Monitoring Reveals Dynamic Levels of T Cell Immunity to Mycobacterium Tuberculosis in HIV Infected Individuals. PLoS ONE; 7:e37920 doi: 10.1371/journal.pone.0037920 PMID: 22685549
12. Kannanganat S, Ibegbu C, Chennareddi L, Robinson HL, Amara RR. Multiple-cytokine-producing antiviral CD4 T cells are functionally superior to single-cytokine-producing cells. J Virol 2007; 81:8468–76 PMID: 17553885
13. Chevalier MF, Julg B, Pyo A, Flanders M, Ranasinghe S, Soghoian DZ, et al. HIV-1-Specific Interleukin-21+ CD4+ T Cell Responses Contribute to Durable Viral Control through the Modulation of HIV-Specific CD8+ T Cell Function. Journal of Virology; 85:733–741 doi: 10.1128/JVI.02030-10 PMID: 21047960
14. Kaufmann DE, Walker BD. PD-1 and CTLA-4 inhibitory cosignaling pathways in HIV infection and the potential for therapeutic intervention. Journal of immunology (Baltimore, Md: 1950) 2009; 182:5891–7 doi: 10.4049/jimmunol.0803771 PMID: 19414738
15. Garrait V, Cadranel J, Esvant H, Herry H, Morinet P, Mayaud C, et al. Tuberculosis generates a microenvironment enhancing the productive infection of local lymphocytes by HIV. Journal of immunology 1997; 159:2824–30 PMID: 9300705
